# Genome-wide microarray analysis of tomato roots showed defined responses to iron deficiency

**DOI:** 10.1186/1471-2164-13-101

**Published:** 2012-03-20

**Authors:** Anita Zamboni, Laura Zanin, Nicola Tomasi, Mario Pezzotti, Roberto Pinton, Zeno Varanini, Stefano Cesco

**Affiliations:** 1 Department of Biotechnology, University of Verona, via delle Grazie 15, 37134 Verona, Italy; 2 Department of Agriculture and Environmental Sciences, University of Udine, via delle Scienze 208, 33100 Udine, Italy; 3 Faculty of Science and Technology, Free University of Bolzano, piazza Università 5, 39100 Bolzano, Italy

## Abstract

**Background:**

Plants react to iron deficiency stress adopting different kind of adaptive responses. Tomato, a *Strategy I *plant, improves iron uptake through acidification of rhizosphere, reduction of Fe^3+ ^to Fe^2+ ^and transport of Fe^2+ ^into the cells. Large-scale transcriptional analyses of roots under iron deficiency are only available for a very limited number of plant species with particular emphasis for *Arabidopsis thaliana*. Regarding tomato, an interesting model species for *Strategy I *plants and an economically important crop, physiological responses to Fe-deficiency have been thoroughly described and molecular analyses have provided evidence for genes involved in iron uptake mechanisms and their regulation. However, no detailed transcriptome analysis has been described so far.

**Results:**

A genome-wide transcriptional analysis, performed with a chip that allows to monitor the expression of more than 25,000 tomato transcripts, identified 97 differentially expressed transcripts by comparing roots of Fe-deficient and Fe-sufficient tomato plants. These transcripts are related to the physiological responses of tomato roots to the nutrient stress resulting in an improved iron uptake, including regulatory aspects, translocation, root morphological modification and adaptation in primary metabolic pathways, such as glycolysis and TCA cycle. Other genes play a role in flavonoid biosynthesis and hormonal metabolism.

**Conclusions:**

The transcriptional characterization confirmed the presence of the previously described mechanisms to adapt to iron starvation in tomato, but also allowed to identify other genes potentially playing a role in this process, thus opening new research perspectives to improve the knowledge on the tomato root response to the nutrient deficiency.

## Background

Iron (Fe) deficiency is a yield-limiting factor for a variety of field crops all around the world and generally results from the interaction of limited soil Fe bioavailability and susceptible genotype cultivation [1]. Iron is an important microelement for plant life due to its involvement as redox-active metal in photosynthesis, mitochondrial respiration, nitrogen assimilation, hormone biosynthesis, production and scavenging of reactive oxygen species, osmoprotection and pathogen defence [2].

Under aerated conditions at neutral alkaline pH, the soluble Fe concentration in soil solution is very low. To cope with Fe shortage plants have developed two strategies for its acquisition. The *Strategy I *(all higher plants except grasses) relies on improvement of Fe uptake through acidification of soil solution by excretion of protons via a plasmalemma P-type ATPase resulting in an increased Fe solubility, reduction of Fe^3+ ^to the more soluble Fe^2+ ^by a Fe^III^-chelate reductase and plasmalemma transport of Fe^2+ ^by the activity of a Fe transporter [3]. Some model plants used to study *Strategy I *are dicots such as *Arabidopsis thaliana, Solanum lycopersicum *and *Pisum sativum *[4].

Plant responses to Fe deficiency have been recently analyzed on the basis of large-scale changes not only in transcriptome [5-14], but also in proteome [15-20] and metabolome [17]. Results of transcriptome analysis are influenced by differences in experimental plans, plant species and microarray platforms, and thus difficult to compare and be generalized. Notwithstanding this drawback, recently, a set of 92 transcripts that robustly reflect the transcriptional response of *Arabidopsis *to Fe deficiency [21], has been described as the "ferrome" by Schmidt and Buckhout [21]. The "ferrome" consists of a list of transcripts considered to be involved in the basic response to iron deficiency. The ferrome is particularly enriched in genes related to heavy metal cation transport and metal homeostasis. Focusing on tomato, a plant often used as a model to study Fe deficiency (*Strategy I*) and a crop of economic importance, no information is available at genome-wide transcriptional level. Two proteomic characterizations of tomato roots in response to 1-week of Fe deprivation showed 23 [15] and 15 [16] differentially expressed protein spots respectively. Modifications in proteome suggest changes in energy metabolism, sulfur metabolism, response to oxidative stress and signal transduction.

In the present work a genome-wide transcriptional characterization of tomato roots in response to Fe deficiency is presented. This approach allowed indentifying 97 differentially expressed transcripts involved in the responses to the nutritional stress. Transcriptional changes, mainly related to positive modulation of glycolysis, TCA and methionine cycle, suggest that tomato roots behave similarly to *Arabidopsis *under Fe deficiency. Furthermore, flavonoid biosynthesis and root morphological changes are revealed as specific tomato responses to Fe shortage.

## Results and discussion

### Responses to Fe-deficiency

**Figure 1:**
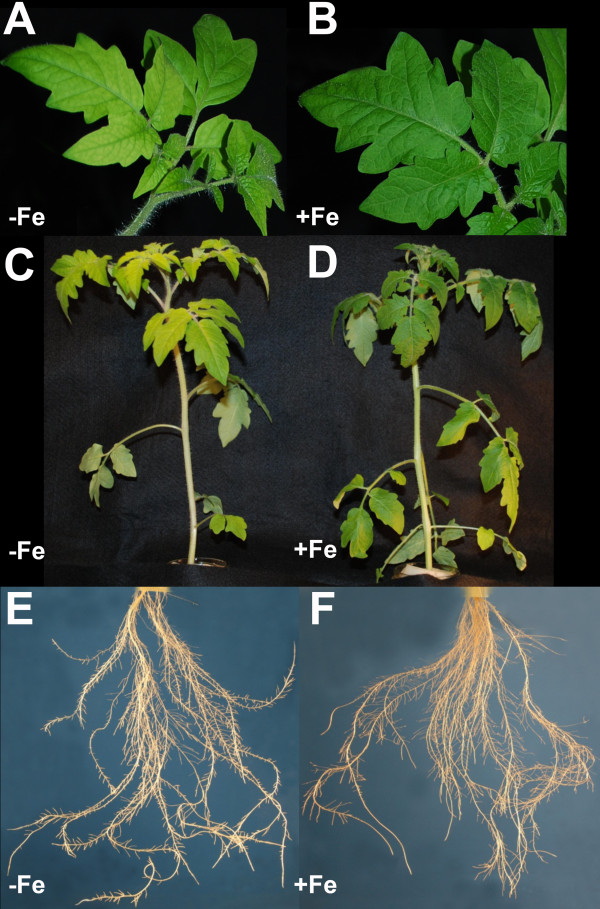
**Shoot and root apparatus of tomato plants grown under different Fe-supply conditions**. Leaf detail of Fe-deficient (**A**) and Fe-sufficient (**B**) plants. Shoot (**C**) and roots (**E**) of Fe-deficient plants and shoot (**D**) and roots (**E**) of Fe-sufficient plants.

**Figure 2:**
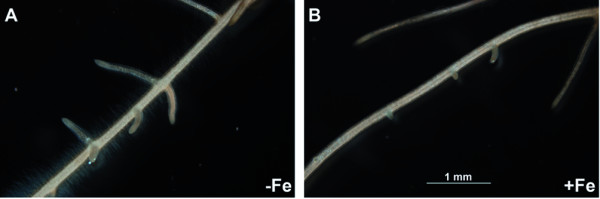
**Root apparatus of tomato plants grown under different Fe-supply condition**. Detail of root apparatus of **A**) Fe-deficient and **B**) Fe-sufficient plants.

Typical responses of Fe-deficiency [22] were observed in tomato plants grown for 14 d in the presence of a low amount of Fe and thereafter subjected to 7 d of Fe deprivation. The chlorophyll content (SPAD index value) was reduced in Fe-deficient plants (Table 1). A concomitant increase in root Fe^III^-chelate reductase activity (Table 1) was also observed with values similar to those commonly found in roots of Fe-deficient tomato plants [23]. Furthermore, Fe-deprived tomato plants developed more lateral roots and showed an abundant production of root-hairs (Figures 1 and 2).

**Table 1:** Leaf SPAD index values and root Fe^III^-chelate reductase activity

Sample	**SPAD index**^**a**^	**Fe^III^-chelate reductase (mol g^-1 ^root FW h^-1^)**^**b**^
Fe-sufficient	29.5 ± 0.3	0.37 ± 0.04

Fe-deficient	16.8 ± 0.6	1.41 ± 0.06

^a^SPAD index value of fully expanded young leaves was determined using a SPAD-502 meter (Minolta, Osaka, Japan); mean and SD using data of the three biological replicates.

^b^Mean and SD of three biological replicates.

### Comparison of root gene expression profiles in Fe-deficient and Fe-sufficient tomato plants

Differences in root gene expression between Fe-sufficient and Fe-deficient tomato plants were obtained by a genome-wide gene expression analysis using a tomato chip developed through Combimatrix technology [24]. This chip allows monitoring simultaneously the expression of more than 25,000 tomato transcripts. Ninety-seven differentially expressed transcripts between Fe-deficient and Fe-sufficient tomato roots (75 up-regulated and 22 down-regulated) were identified by Linear Models for MicroArray (LIMMA) [25] (adjusted p-value ≤ 0.05; |FC| ≥ 2). This result obtained using a large-scale chip reinforce the idea that plant transcriptional response to Fe shortage is based on the modulation of a relative small set of genes as previously observed for the *Arabidopsis *"ferrome" [21].

**Figure 3:**
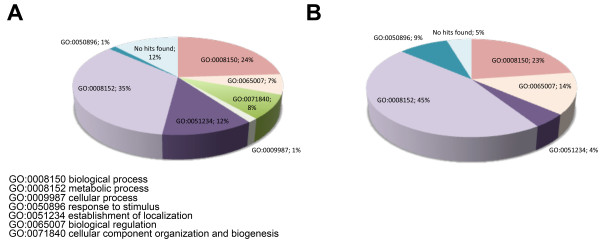
**Functional categories distribution of differentially expressed transcripts**. Distribution in main functional categories according to the GO "biological process" terms of the 75 up-regulated (**A**) and 22 down-regulated (**B**) transcripts in roots of Fe-deficient relative to Fe-sufficient plants. Percentage of transcripts is reported for each functional category.

Manually curated annotation of the 97 differentially expressed transcripts was based on results of BlastP analysis against UniProt [26] database (Figure 3; Additional file 1) using terms of biological process of Gene Ontology (GO) [27]. Sequence grouping in functional categories according to the GO terms revealed that the most abundant functional category was "metabolic process" both for up-regulated and down-regulated transcripts (35% and 45% respectively; Figure 3). Other up-regulated transcripts belonged to "establishment of localization" (12%) and "cell wall organization and biogenesis" (8%), while for the down-regulated transcripts "response to stimulus" (9%) was one of the most representative main functional categories (Figure 3). Only up-regulated transcripts are present in the "secondary metabolic process" category (Table 2). Transcripts encoding proteins with no sequence homology to known proteins were defined as "no hits found" (12% and 5% for up-regulated and down-regulated transcripts respectively), while a similar percentage of transcripts showed homology to proteins involved in "unknown" biological process (24% and 23% respectively). A selection of differentially expressed and discussed in relation to Fe deficiency is reported in Table 2. The up-regulation and down-regulation of six differentially expressed transcripts in response to Fe deficiency were confirmed through Real-time RT-PCR experiments (Table 3).

**Table 2:** List of transcripts modulated in response to Fe-deficiency and reported in the Discussion

#	**ProbeID**^**a**^	**Description**^**b**^	**UniProtID**^**c**^	**TC-ID**^**d**^	**FC**^**e**^	***p*, value, adj**^**f**^
	*Biological process GO:0008150*					
*1*	TC192724_853_37_S	**SRC2**	B6SND5	TC192724	3.02	0.014
*2*	TC192763_241_40_S	*Putative uncharacterized protein*	C6T3H9	TC192763	2.11	0.030
*3*	TC193319_801_34_X2	*Putative uncharacterized protein*	Q40127	TC193319	-3.35	0.016
*4*	TC195341_998_35_S	*Putative uncharacterized protein*	Q40127	TC195341	-3.33	0.011
*5*	TC196753_1279_37_S	*D-protein*	Q8VWY8	TC196753	6.11	0.028
*6*	TC197095_638_34_X2	*NtEIG-E80 protein (elicitor inducible gene product)*	Q9FXS6	TC197095	4.69	0.024
*7*	TC198323_947_40_S	*Predicted protein*	B9MWQ1	TC198323	2.51	0.032
*8*	TC199253_1439_39_S	*Zinc finger protein. putative*	B9SLY6	TC199253	2.82	0.029
*9*	TC202360_487_40_S	**EF-1 alpha-like protein**	O49604	TC202360	2.15	0.024
*10*	TC204571_463_40_S	*ATBET12, putative*	B9STJ3	TC204571	-2.62	0.023
*11*	TC205207_890_35_S	*Putative uncharacterized protein*	Q40127	TC205207	-3.09	0.048
*12*	TC207200_893_35_S	*VIT_00038707001*	E0CVH7	TC207200	2.44	0.042
*13*	TC207407_739_37_S	*Predicted protein*	B9HQW6	TC207407	2.36	0.042
*14*	TC207665_362_36_S	*Putative uncharacterized protein*	A9PCS8	TC207665	-2.39	0.048
*15*	TC208712_583_40_S	**Germin-like protein**	Q5DT23	TC208712	2.35	0.046
*16*	TC208745_692_37_S	*Putative uncharacterized protein*	A5C0F7	TC208745	4.84	0.016
*17*	TC209321_482_35_S	*VITISV_041870*	A5C9V2	TC209321	6.94	0.011
*18*	TC209504_302_40_S	*Hydrolase*	Q4PSL3	TC209504	2.78	0.013
*19*	TC211515_728_35_S	*Amino acid binding protein, putative*	B9RBU8	TC211515	3.66	0.014
*20*	TC212954_1137_35_S	*Predicted protein*	B9HZ36	TC212954	2.02	0.046
*21*	TC213456_100_34_S	*Putative D-protein*	Q6K482	TC213456	4.97	0.035
*22*	TC214599_1004_37_S	**Nodulin-like proteinAt2g16660/T24I21.7**	Q9SLF1	TC214599	6.87	0.042
*23*	TC215994_489_36_S	*Putative aminotransferase, class V family protein*	Q1H8R9	TC215994	2.08	0.036
	*Biological regulation GO: 0065007*					
*24*	TC191806_907_40_S	**BHLH transcriptional**	Q5GA67	TC191806	6.56	0.024
*25*	TC191963_1058_37_S	**Ferritin**	Q308A9	TC191963	-4.71	0.008
*26*	TC194645_664_37_S	**DNA binding protein**	B9SZX2	TC194645	12.34	0.015
*27*	TC198138_1325_39_S	**Thioredoxin peroxidase 1**	Q7Y240	TC198138	7.06	0.046
*28*	TC203853_451_35_S	**Heterogeneous nuclear ribonucleoprotein 27C**	B9SSS5	TC203853	2.90	0.028
*29*	TC206202_500_40_S	**Thioredoxin II**	B9RLX0	TC206202	-2.47	0.048
*30*	TC215976_330_38_X2	**Ferritin**	Q308A9	TC215976	-3.64	0.005
	*Cellular component organization or biogenesis GO:0071840*					
*31*	NP000231_1302_40_S	**Extensin-like protein Dif54**	Q43505	NP000231	8.22	0.011
*32*	TC191669_1238_40_S	**Extensin-like protein Ext1**	Q8VWM5	TC191669	5.53	0.003
*33*	TC204863_245_40_S	**Extensin-like protein Ext1**	Q8VWM5	TC204863	8.98	0.013
*34*	TC212258_415_40_S	**Extensin-like protein Dif54**	Q43505	TC212258	9.13	0.011
*35*	TC212487_279_40_S	**Extensin-like protein Ext1**	Q8VWM5	TC212487	6.44	0.0004
*36*	TC214133_1133_40_S	**Extensin-like protein Dif10**	Q43504	TC214133	5.42	0.024
	*Cellular process GO:0009987*					
*37*	TC207486_546_40_S	**Pollen specific actin-depolymerizing factor 2**	Q8H2B6	TC207486	4.81	0.005
	*Establishment of localization GO: 0051234*					
*38*	NP796451_1558_36_S	**Root-specific metal transporter**	Q84LR1	NP796451	12.00	0.024
*39*	TC191581_1150_36_S	**Iron-regulated transporter 1**	Q9XFB2	TC191581	9.42	0.013
*40*	TC192292_1560_39_S	**Hippocampus abundant transcript 1 protein**	B9SG70	TC192292	14.79	0.0004
*41*	TC200857_1001_40_S	**Ammonium transporter 1 member 1**	P58905	TC200857	2.05	0.043
*42*	TC205660_580_35_S	**Metal tolerance protein**	B9GLJ8	TC205660	3.77	0.049
*43*	TC206149_966_36_S	**Aluminum-activated malate transporter 9**	Q9LS46	TC206149	2.41	0.048
*44*	TC208376_922_36_S	**Oligopeptide transporter, putative**	B9SA63	TC208376	17.67	0.004
*45*	TC215768_1111_35_X2	**Aquaporin**	Q8W506	TC215768	-2.03	0.023
*46*	TC215874_553_40_S	*Sec14 cytosolic factor, putative*	B9S6A7	TC215874	3.5	0.009
*47*	TC216882_1121_38_X2	**Hippocampus abundant transcript 1 protein**	B9SG70	TC216882	9.29	0.003
	*Metabolic process GO:0008152; Carbon utilization GO:0015976*					
*48*	TC204225_1412_35_S	**Phosphoenolpyruvate carboxylase**	P27154	TC204225	3.31	0.039
*49*	TC214978_700_36_S	**Phosphoenolpyruvate carboxylase**	P27154	TC214978	3.74	0.036
	*Metabolic process GO:0008152; Catabolic process GO:0009056*					
*50*	TC194584_1854_37_S	**Cysteine-type peptidase, putative**	B9STX0	TC194584	3.61	0.011
*51*	TC203350_1573_40_S	**Vacuolar processing enzyme 1**	B2M1T0	TC203350	2.18	0.026
*52*	TC208154_2059_38_S	**Beta-amylase PCT-BMYI**	Q94EU9	TC208154	-3.06	0.042
*53*	TC215970_3405_40_S	**Protease Do-like 7**	Q8RY22	TC215970	4.01	0.032
	*Metabolic process GO:0008152; Cellular metabolic process GO:0044237*					
*54*	TC192049_1645_40_S	**Sulfate adenylyltransferase**	Q43183	TC192049	-2.48	0.029
*55*	TC193934_828_38_X2	**Methylthioribose kinase, putative**	B9RY82	TC193934	2.12	0.023
*56*	TC194380_182_36_S	*S-adenosylmethionine-dependent methyltransferase, putative*	B9SZS6	TC194380	2.03	0.045
*57*	TC195032_699_35_S	**CBL-interacting serine/threonine-protein kinase 11**	O22932	TC195032	-2.62	0.030
*58*	TC198109_708_34_X2	**Phosphofructokinase, putative**	B9RRX6	TC198109	2.00	0.036
*59*	TC199972_1164_40_S	**Fructose-bisphosphate aldolase**	Q2PYX3	TC199972	2.53	0.022
*60*	TC201350_645_36_S	**Protein phosphatase-2C**	O82469	TC201350	2.10	0.028
*61*	TC201692_547_40_S	**Xyloglucan endotransglucosylase/hydrolase 14**	B9RKL5	TC201692	7.29	0.013
*62*	TC206357_361_38_S	**Catalase isozyme 1**	P30264	TC206357	-2.79	0.046
*63*	TC212978_951_34_S	**Phosphoenolpyruvate carboxylase**	Q8S915	TC212978	3.62	0.030
*64*	TC214826_1684_35_S	*ATP binding protein, putative*	B9RII2	TC214826	12.39	0.011
*65*	TC214837_697_40_S	**Cytokinin oxidase/dehydrogenase**	C3VPM8	TC214837	4.64	0.015
*66*	TC216529_896_39_S	**Peroxidase 7**	Q9SY33	TC216529	9.25	0.011
*67*	TC216572_542_40_S	**Avr9/Cf-9 rapidly elicited protein 216**	Q84QE0	TC216572	-2.61	0.042
	*Metabolic process GO:0008152; Oxidation reduction GO:0055114*					
*68*	TC191412_2284_38_S	**Ferric-chelate reductase**	Q6EMC0	TC191412	15.21	0.008
*69*	TC191893_9_37_S	**Superoxide dismutase**	Q7YK44	TC191893	-2.29	0.030
*70*	TC194139_2083_38_S	**Ferric-chelate reductase**	B9RIU2	TC194139	-10.76	0.023
*71*	TC194227_76_41_X2	**Superoxide dismutase**	Q7YK44	TC194227	-2.20	0.038
*72*	TC196465_645_40_X2	**Gibberellin 20 oxidase**	B9RUX2	TC196465	3.46	0.009
*73*	TC199400_1132_37_S	**Peroxidase 2, putative**	B9SZA0	TC199400	-2.05	0.022
*74*	TC201832_1136_40_S	**Gibberellin 20 oxidase**	B9RUX2	TC201832	3.00	0.013
*75*	TC205699_961_40_S	*Chlorophyll synthase, putative*	B9RJ38	TC205699	7.12	0.019
*76*	TC207549_14_41_S	**Cytochrome C oxidase polypeptide vib**	B9RJN9	TC207549	3.05	0.016
*77*	TC208767_662_38_S	**Cationic peroxidase 1**	B9SWU3	TC208767	4.75	0.022
*78*	TC213071_429_40_S	**Peroxidase**	Q07446	TC213071	-3.29	0.032
	*Metabolic process GO:0008152; Primary metabolic process GO:0044238*					
*79*	TC197609_880_35_S	*Zinc finger protein. putative*	B9T6Q0	TC197609	3.54	0.014
*80*	TC192838_1967_36_S	**Endo-1,4-beta-glucanase**	B9RLZ9	TC192838	2.45	0.042
	*Metabolic process GO:0008152; Secondary metabolic process GO:0019748*					
*81*	TC197109_626_37_S	**Flavonoid 3-hydroxylase, putative**	B9T1C6	TC197109	2.56	0.024
*82*	TC198786_1057_37_S	**UDP-glucose:glucosyltransferase**	B6EWY6	TC198786	2.19	0.022
*83*	TC203267_704_38_S	**UDP-glucose:flavonoid glucoside 1,6-glucosyltransferase**	C5NN14	TC203267	2.76	0.013
*84*	TC212095_566_35_S	**UDP-glucose:flavonoid glucoside 1,6-glucosyltransferase**	C5NN14	TC212095	3.69	0.018
	*Response to stimulus GO:0050896*					
*85*	TC193192_66_41_X2	**Peroxidase 4**	B7UCP4	TC193192	-3.86	0.029
*86*	TC208216_282_40_S	*Pit1 protein*	Q40539	TC208216	4.71	0.019
*87*	TC195700_1019_40_S	**Peroxidase**	B9VRK9	TC195700	-2.67	0.013
	*No hits found*					
*88*	TC203837_663_35_S	*No hits found*		TC203837	2.06	0.046
*89*	TC204355_604_36_S	*No hits found*		TC204355	-3.09	0.038
*90*	TC207055_310_37_S	*No hits found*		TC207055	3.05	0.030
*91*	TC209134_260_40_S	*No hits found*		TC209134	30.06	0.007
*92*	TC209988_335_40_S	*No hits found*		TC209988	6.82	0.013
*93*	TC211287_216_38_S	*No hits found*		TC211287	7.63	0.011
*94*	TC212074_241_40_S	*No hits found*		TC212074	6.44	0.023
*95*	TC212933_306_37_S	*No hits found*		TC212933	9.59	0.011
*96*	TC214074_254_40_S	*No hits found*		TC214074	2.22	0.041
*97*	TC215128_252_35_S	*No hits found*		TC215128	3.86	0.029

^a^ID of TomatoArray2.0 probes

^b^Description of each transcript. Bold discussed; italics undiscussed.

^c^UniProtID [26] of the first hit obtained by BlastP analysis

^d^ID of the TC of DFCI Tomato Gene Index (Release 12.0) [92]

^e^Fold change value

^f^adjusted *p*-value

**Table 3:** Real-time RT-PCR validation of a set of genes differentially expressed in microarray analysis

TC ID	Description	Real-time RT-PCR (*ratio*)	Microarray
TC208376	Oligopeptide transporter, putative	17.87 ± 4.35	17.67

NP796451	Root-specific metal transporter	15.11 ± 3.97	12.00

TC191581	Iron-regulated transporter 1	3.07 ± 0.59	9.42

TC216882	Hippocampus abundant transcript 1 protein, putative	8.69 ± 1.53	9.29

TC205660	Metal tolerance protein	1.55 ± 0.44	3.77

TC194139	Ferric-chelate reductase, putative	-12.14 ± 3.15	-10.76

*TC ID*, description, Real-time *RT-PCR *relative expression value (Fe-deficient vs. Fe-sufficient) and microarray fold change value (Fe-deficient vs. Fe-sufficient). Real time data were normalized on the *EF1a *gene and were performed 4 times on 3 independent experiments. Real-time RT-PCR data are expressed as mean ± SD.

Sixty-one of 97 transcripts are ascribable to adaptive responses to Fe deficiency involving Fe homeostasis, metabolic process, oxidative stress responses, root morphological modification, transport processes, hormone metabolism and signaling. The others are hardly related to specific role showing homology to protein without a specific biological process or lacking homology to known protein ("no hits found").

### Fe homestasis

Our transcriptional analysis confirmed that roots of Fe-deficient tomato plants overexpressed genes involved in Fe uptake and reduction, including the transcripts encoding IRON-REGULATED TRANSPORTER (IRT) [28-30] and Fe^III^-chelate reductase (FRO) [31]. The tomato bHLH protein (encoded by *LeFER*) plays a role in Fe-deficiency responses through the expression of these two tomato Fe mobilization genes belonging to the Fe uptake systems of the *Strategy I *plants [29,32,33]. Our data show the up-regulation of the *FER *transcript (#*24*) in Fe-deficient roots, which is in agreement with the positive modulation of Fe-uptake-related transcripts such as *LeFRO1 *(#*68*), *LeIRT1 *(#*39*) and a transcript encoding a NATURAL RESISTANCE-ASSOCIATED MACROPHAGE PROTEIN1 (LeNRAMP1; #*38*) [29,32]. A positive modulation of FER and Fe mobilization proteins (IRT1, NRAMP1 and FRO1) was not found in two proteomics studies performed in tomato roots grown in conditions similar to those used in the present work [15,16]. Authors justified these results as related to the features of proteomic approach, which was not sensitive enough to detect FER and not well suited for membrane-bound proteins. However, functional characterizations of *fer *mutant proved that FER controls the expression of the iron-uptake genes [29,32,33]. A transcriptional behaviour similar to that described in the present work was observed for *Arabidopsis *orthologous genes in Fe-starved roots [5,8,11,34].

Our data show a strong down-regulation of another ferric chelate reductase (#*70*). Previous results indicated that the same transcript (TC194139 of the Release 12.0, corresponding to TC124302 of the Release 9.0) specific to the *Solanum lycopersicum *genome, is only slightly regulated by Fe and that its function is not essential for Fe uptake [34]. Our results showing a negative regulation of this FRO transcript in response to Fe-deficiency in roots, also quantified by Real-time RT-PCR experiment, confirm that this gene does not play a crucial role in deficiency-induced Fe uptake and suggest the involvement in other biological process.

Together with the positive modulation of *LeFER, LeFRO1 *and *LeIRT1*, we observed for the first time a high up-regulation of another bHLH transcript (#*26*); this result suggests that like in *Arabidopsis *[35] also in tomato plants the response to Fe deficiency through FER activity may need the interaction with another bHLH protein. BlastP analysis against TAIR database [36] using protein sequence obtained from the predicted coding sequence of the TC194645 showed the highest sequence homology with the protein encoded by *AtbHLH38 *(score: 120; Evalue: 9 E-28; identity: 35%; positives: 57%) known to interact with FIT, the *Arabidopsis *orthologous of tomato FER [35].

The down-regulation of two ferritin transcripts (#*25 *and *30*) is in line with the negative regulation of ferritin genes observed in roots of Fe-depleted *Arabidopsis *plants [5,8,11]. It has been suggested that ferritins can be involved in Fe homeostasis [37] with a main role of plastidial Fe [11]. *Arabidopsis *nodulin-like genes have been recently described to be putatively involved in Fe transport and storage under metal cation sufficiency [38]. Transcriptional levels of *Arabidopsis *nodulin-like genes were down-regulated at least until 72-h of Fe-deficiency, while two other nodulin-like genes were not modulated in response to different Fe conditions [38]. On the other hand in tomato roots we recorded a positive modulation of two transcripts encoding protein with a nodulin-like domain (#*9 *and *22*). BlastP analysis against TAIR database [36] using protein sequence obtained from the predicted coding sequence of the TC214599 (#*22*) showed the highest sequence homology with the protein encoded by At3g43660 (score: 325; Evalue: 2 E-29; identity: 54%; positives: 68%) which is one of the two *Arabidopsis *genes not modulated by iron [38].

### Metabolic processes

#### Carbohydrate metabolism

As observed in *Arabidopsis *root microarray analyses [5,8,11] glycolysis-related genes are positively modulated in Fe-deficient roots. These transcriptional data fit well with the increased activity of glyceraldehyde-3-phosphate dehydrogenase (GADPH), pyruvate kinase (PK), and phosphofructokinase (PFK) recorded in response to Fe starvation in cucumber roots [39]. Furthermore, increased levels of protein related to glycolysis under Fe shortage were recently reported in sugar beat [17] and *Medicago truncatula *[20] roots. All together these evidences are consistent with the idea of a shift from anabolic to catabolic metabolism. In the present work, a fructose-bisphosphate aldolase (FBP aldolase) (#*59*) and a PFK (#*58*) transcripts were up-regulated under Fe deficiency, further confirming changes in primary metabolism in response to Fe starvation. The positive modulation of transcript encoding a PFK, an enzyme catalysing a protogenic reaction, supports the role of glycolysis in different process such as production of ATP and H^+ ^for H^+^-ATPase, reducing equivalents for ferric chelate reductase and of phosphoenolpyruvate (PEP) [40]. Up-regulation of three phosphoenolpyruvate carboxylase (PEPC) transcripts (#*48, 49 *and *63*) agrees with results of several proteomic and physiological studies on the response to Fe deficiency in tomato [15,16,41], sugar beat [17] and *Medicago truncatula *roots [20], showing a positive modulation of proteins involved both in glycolysis and TCA cycle. PEPC activity, through pyruvate consumption, can keep active the glycolytic pathway and give a contribution to the control of cytosolic pH [40,41]. In Fe deficiency, starch catabolism was reported to be enhanced both at transcriptional [5] and protein level [16]. Our analysis revealed a down-regulation of a transcript encoding a protein showing homology to a potato chloroplastic ß-amylase (#*52*) involved into starch degradation in plastids [42]. Since starch catabolism mediated by this enzyme occurs in plastids, the negative modulation of ß-amylase could be ascribed to other causes than an accelerated glycolysis.

The positive modulation of a cytochrome C oxidase (#*76*) is in line with the transient induction of electron-transport-chain genes observed in *Arabidopsis *[5] and consistent with the enhancement of respiration rate observed in cumber [39] and sugar beat [43] Fe-deficient roots. This behaviour was interpreted as an attempt to increase energy production through oxidative phosphorylation. However, more recently it has been suggested that the increased respiration rate in root segments of Fe-starved cucumber plants should not be interpreted as an increase in mitochondrial activity but rather as the result of an increase in the number of less efficient mitochondria and of the induction of different O_2_-consuming reaction [44].

#### Methionine cycle

Nicotianamine (NA) is considered to be a key molecule for long-distance transport of Fe in plants [45]. Proteomic analysis of Fe-starved tomato roots [16] showed a positive modulation of proteins related to metabolism of methionine (e.g. methionine synthase), a precursor of nicotianamine. An up-regulation of a transcript encoding a methylthioribose kinase (MTK, #*55*), another enzyme of the methionine cycle, was observed in our transcriptional analysis. A positive modulation of MTK transcripts was also recorded in roots of rice, a *Strategy II *plant species, under Fe deficiency [46,47], although in this case, related to the necessity to increase the production of mugineic acid.

A down-regulation of a sulfate adenylyltransferase (ATPS; ATP sulfurylase) gene (#*54*) was observed in this work. However, the opposite was found in *Arabidopsis *[11]. The connection between the sulphur nutritional status and capability to respond to Fe shortage has been demonstrated in both *Strategy I *and *Strategy II *plants [30,48]. Recently an increase in methionine content related to phytosiderophore synthesis without significant changes in ATPS activity was described in Fe-deficient barley roots [48]. Interestingly, in a recent study of *Medicago truncatula *root proteome [20] two enzymes related to biosynthesis of cysteine and methionine were negatively affected by Fe deficiency.

#### Protein turnover

Response to Fe starvation induced the accumulation of gene transcripts related to protein turn-over, including protease (#*53*) and peptidase (#*50 *and *51*) involved in protein degradation, and a gene encoding a heterogeneous nuclear ribonucleoprotein (#*28*) that can act in the pre-mRNA metabolism preceding protein synthesis.

The activity of these genes can be related to molecular events controlling plant responses to abiotic stresses such as nutritional deficiencies [49]. A general increase in protein synthesis was reported as a response to Fe-deficiency in cucumber roots [50]; furthermore, protein recycling in response to Fe starvation was suggested by analysis of expression profiles of soybean [13] and proteomic changes in cucumber [18] and *Medicago troncatula *[20] roots. Transcriptomic data presented here are in line with the idea that, under Fe deficiency, N recycling reactions take place, possibly related to the necessity of additional anaplerotic source of C and N [18,20].

#### Secondary metabolism

Phenolic compounds are reported as components of root exudates in Fe-deficient *Strategy I *plants. These molecules are involved in chelation and/or reduction of rhizospheric insoluble Fe [51,52]. Recently, phenolics have been proposed to selectively influence rhizospheric microorganisms and be involved in the reutilization of apoplastic Fe [53]. Our results showed an up-regulation of a flavonoid-3-hydroxylase (#*81*) gene and of three genes putatively involved into flavonoid glycosylation (#*82, 83 *and *84*). Two out of the last three genes showed sequence homology to a *Catharanthus roseus *flavonoid glucoside 1,6-glucosyltransferase catalysing 1,6-glucosylation of flavonol and flavone glucosides [54]. A positive modulation of genes related to general phenylpropanoid pathway (e.g. genes encoding PAL and 4CL) was reported in Fe-deficient *Arabidopsis *roots [11]. Data of the present work underline the up-regulation of transcripts involved in a more specific branch of phenolic pathway supporting the idea that Fe-starved roots might operate flavonoid secretion into the rhizosphere in order to promote Fe acquisition [55].

### Oxidative stress responses

Many proteins involved in antioxidative defence response contain Fe in heme group or coordinated to the thiol group of cysteine. The modulation of transcripts and proteins related to oxidative stress response in roots of Fe-starved plants seems to depend on the species and the experimental conditions [5,13,17,19]. However, catalase (CAT) and peroxidase (POX) activities are known to be depressed under Fe deficiency conditions [56] in tomato leaves. Our data showed a main down-regulation of transcripts encoding thioredoxin (TRX) (#*29*) and detoxifying enzymes catalase (CAT; #*62*), superoxide dismutase (#*69 *and 71), peroxidase (POX; #*73, 78, 85 *and *87*). Two other POX transcripts (#*66 *and *77*) and a thioredoxin peroxidase gene (#*27*) were, conversely, up-regulated. At protein level, a decrease of a CAT was reported while some POXs showed higher levels in response to Fe deficiency in tomato roots [15]; a different response of peroxidase isoforms has also been reported in sunflower [57]. Taken together, data of the present work suggest a different role in response to nutrient stress condition between the thioredoxin (ABB) and POX isogenes and are in agreement with previous results obtained in *Medicago truncatula *[20] and sugar beat [17] Fe-deficient roots.

A germin-like transcript (#*15*) was up-regulated under Fe deficiency, similarly to what has been observed in the tomato root proteome analysis [15]. The positive modulation of a germin protein reported in this proteomic study was justified hypothesizing its role in producing hydrogen peroxide for apoplastic Fe reduction or in other stress response on the basis of sequence similarity to a *Nicotiana attenuata *germin protein [15,58].

### Root morphological adaptation

Morphological modifications in roots of Fe-deficient plants are well documented [59,60]. Root hairs proliferation and development of transfer cells were described in Fe-starved tomato plants [29,61-63]. Enhanced formation of lateral roots and root hairs was also recorded in our experiment (Figure 1). Extensin proteins seem to be involved in this latter phenomenon; in fact, we observed a strong positive modulation of LeExt1 (#*32, 33 *and *35*), LeDif10 (#*36*) and LeDif54 (#*31 *and *34*). It was reported that these three genes encode extensin, a structural protein putatively conferring physical characteristics of the cell wall [64], and act during root hair formation in tomato due their predominant expression in root hair cells [64,65].

The overexpression of an endo-1,4-β-glucanase (#*80*) and a xyloglucan endotransglucosylase/hydrolase protein (#*61*) transcripts could be involved into the cell wall loosening [66] associated to root morphological adaptation to Fe deficiency, as previously observed in a tomato root proteomic analysis [16]. These changes in root morphology are also supported by the up-regulation of a transcript showing homology to a tobacco actin-depolymerizing factor (#*37*) related to the pollen tube elongation [67]. Here, we report a negative modulation of a transcript (#*45*) showing homology to the tobacco aquaporin PIP2 [68] suggesting a role of the PIP2 tomato protein in Fe-related morphological root changes. It has been suggested that PIP aquaporins can play a role not only in root water uptake but also in root development [68]. Transgenic plants exhibiting RNAi of PIP2 aquaporins showed a significant increase in the length of primary roots [68].

### Transport processes

Among the positively modulated transcripts belonging to "transport" functional category, a stronger modulation (more than 17 times) of an oligopeptide transporter (OTP) gene (#*44*) was observed under Fe shortage. BlastP analysis against TAIR database [36] using protein sequence obtained from the predicted coding sequence of the TC208376 (#*44*) showed the highest sequence homology with the protein encoded by *AtOPT3 *(score: 541; Evalue: E-154; identity: 83%; positives: 90%). Positive modulation of *AtOPT2 *and *AtOPT3 *has been recorded in Fe-deficient *Arabidopsis *roots [8,11,21,69]. The plant members of OTP family have been described to have different functions in transport physiology such as long-distance sulphur distribution, nitrogen mobilization, metal homeostasis, and heavy metal sequestration. OPTs can transport glutathione, metal-chelates and peptides [70]. It was hypothesized that some plants OTPs are able to transport Fe-chelates and Fe-NA suggesting a role of these proteins in long-distance metal transport *in planta *[71,72]. A similar function can be hypothesized for the tomato up-regulated OPT transcript, due to the previously described positive modulation of genes related to NA synthesis (see methionine cycle paragraph).

Another up-regulated gene involved in transport phenomena in response to Fe-deficiency is *LeAMT1 *(#*41*). This tomato gene, firstly isolated from a root hair cDNA library [73] was root-specifically expressed and positively regulated by ammonium (NH_4_^+^) in root hairs [73,74]. This result suggests the presence of a linkage between NH_4_^+ ^uptake and Fe shortage. It has been demonstrated that NH_4_^+^-dependent rhizosphere acidification can improve Fe availability in the rhizosphere [75]. Interestingly, nitrate acquisition is limited under Fe deficiency [20,76].

Furthermore, favouring ammonium uptake with respect to nitrate could reduce competition for reducing equivalent between nitrogen and Fe acquisition. This is also in agreement with the hypothesized N-recycling in Fe-deficient plant roots [20].

Fe-deficient tomato roots strongly overexpressed two transcripts (#*40 *and *47*) encoding a protein sharing features of major facilitator superfamily (MSF) with putative transport activities. However, on the basis of their sequence homology it was not possible to hypothesize an involvement in transport of a specific metabolite or mineral nutrient.

A positive modulation of a gene encoding a metal tolerance protein (MTP; #*42*) was also recorded. A similar behaviour was described in Fe-deficient *Arabidopsis *roots [8,11] and interpreted in the light of low specificity of IRT1 transporters, which can transport different metals into the Fe-deficient plants. MTP genes might therefore play a role in the detoxification of zinc ions taken up absorbed under Fe deficiency conditions [11].

Fe deficiency in tomato induced the expression of a transcript (#*43*) encoding a protein showing sequence homology to *Arabidopsis *vacuolar aluminium-activated malate transporter (ALMT) 9 [77]. An increase in organic acids concentration mainly citric and malic acids in plant roots under Fe starvation has been reported for many plant species [78]. However, a decrease of malate levels was observed in tomato root tips after 15-d of Fe deficiency while higher contents were recorded in leaves and xylem sap [41]. The tomato malate transporter gene might be involved in malate fluxes at intracellular level and/or in long-distance transport *in planta*.

### Hormone metabolism and signaling

The role of plant hormones in the regulation of Fe deficiency responses has been extensively studied [79,80]. As for *Arabidopsis *Col-0 accession, we identified the positive modulation of a methionine cycle gene, MTK gene, that could be related not only to NA synthesis (see above) but also to the recycling of methylthioadenosine during ethylene production [11]. This is in agreement with the hypothesized involvement of ethylene and/or auxin in control of hair root production under Fe deficiency [79]. Furthermore, data of the present work suggest the involvement of other hormones (such as gibberellin and cytokinin) in Fe deficiency response in tomato roots. Concerning gibberellin, we recorded the up-regulation of two gibberellin oxidase 20 (GA20OX) transcripts (#*72 *and *74*). They can regulate root morphological changes through the synthesis of active GA1. Indeed, it has been reported that GAs are related to tomato root growth [81]. In addition, it was shown that the expression of a tomato *SlGA20ox1:GUS *construct in *Arabidopsis *localized also in columella of secondary roots [82], thus suggesting a role in secondary root formation. The same authors also reported that the expression of the construct was positively affected in cotyledons, hypocotyls and roots by benziladenine, a synthetic cytokinin [82]. However cytokinins were described as negative regulators of root Fe uptake mechanism in *Arabidopsis *through a root-growth dependent pathway [83]. Cytokinins could play a similar role also in tomato roots. This hypothesis is supported by the up-regulation of a cytokinin oxidase/dehydrogenase (CKX) gene (#*65*) under Fe starvation. *CKX *genes are involved in regulation of plant growth and development through the control of the cytokinin concentration [84].

Focusing on signal transduction, we observed the up-regulation of a transcript (#*60*) encoding a protein with sequence homology to the MCP5, a protein phosphatase-2C (PP2C) of *Mesembryanthemum crystallinum *[85]. McMCP5 is expressed in roots and is induced in response to salt and drought stresses [85]. The tomato PP2C gene could play a role in signal transduction of stress nutrient condition as Fe starvation. The positive modulation of a gene encoding a SRC2 protein containing a C2 domain (#*1*) suggests a putative involvement of Ca as secondary messenger. Focusing on Ca, in our experiment we also identified a down-regulated gene (#*57*) in Fe-starved roots showing homology to an *Arabidopsis *gene (*AtSOS2*) encoding a CBL-interacting serine/threonine-protein kinase 11. *Arabidopsis *SOS2 interacts with the Ca binding protein SOS3 (SALT OVERLY SENSITIVE 3), thus controlling K and Na homeostasis and the response to salt stress [86,87]. In addition a negative modulation of a gene (#*67*) showing homology to a tobacco Avr/Cf-9 rapidly elicited (ACRE) transcript encoding a PP2C was observed [88]. Our data confirm the results obtained with proteome analysis of tomato roots in response to Fe-deficiency, where changes in the levels of proteins involved in signal transduction were reported [16]. The observed transcriptional changes in tomato roots can be the result of the perception of nutrient stress condition and the following signal transduction.

Taken together, these results suggest that Fe deficiency responses are, at least in part, dependent on hormonal balance modifications possibly resulting from signal perception and transduction.

## Conclusion

Ninety-seven differentially expressed transcripts were identified comparing root transcriptional profiles of Fe-deficient and Fe-sufficient tomato plants. Tomato roots respond to Fe deficiency by modulating the expression of a number of transcripts similar to the model plant *Arabidopsis*. The comparison of tomato Fe-responsive transcript set with the *Arabidopsis *"ferrome" [21], encompassing 92 transcripts that robustly represent the response to Fe shortage, confirms the involvement of the well know homologous key regulatory elements (e.g. bHLHs) controlling the expression of transcripts related to Fe uptake and translocation (e.g. IRT and FRO). As showed by *Arabidopsis *"ferrome" [21], tomato roots modulate transcripts involved in homeostasis of Fe and heavy metal cations (e.g. IRT, NRAMP, MTP, ferritin) and others cation (e.g. AMT). Both plant species require the up-regulation of transcripts related to glycolysis (e.g. PFK) and methionine cycle (e.g. MTK), the latter pathway being putatively linked to NA biosynthesis in response to Fe deficiency. Fe-NA complexes could be transported both in tomato and *Arabidopsis *plants through OPTs during the response to Fe shortage. Here we describe, for the first time, the modulation of a specific branch of phenolic (flavonoids) biosynthesis in response to Fe deficiency. In addition, tomato roots seem to be more characterized by root morphological adaptation, mainly linked to hair root production, as suggested by the strong up-regulation of extensin transcripts.

Therefore, this transcriptional study, while confirming evidence coming from proteomic studies, allowed identifying new putative targets for further functional investigations on the response to Fe deficiency in tomato roots.

## Methods

### Plant material, growth conditions and RNA extractions

Tomato seedling (*Solanum lycopersicum *L., cv. 'Marmande superprecoce' from DOTTO Spa, Italy), germinated for 6 days on filter paper moistened with 1 mM CaSO_4_, were grown for 14 days in a continuously aerated nutrient solution (pH adjusted at 6.0 with 1 M KOH) as reported by Tomasi at al. [22] with 5 μM Fe (Fe-EDTA); thereafter, most of the plants were transferred for a further week to a Fe-free nutrient solution (Fe-deficient) and some tomato plants were transferred for a week to a nutrient solution containing 100 μM Fe-EDTA (Fe-sufficient plants) as control. Nutrient solutions were renewed every three days. The controlled climatic conditions were the following: day/night photoperiod, 16/8 h; light intensity, 220 μE m^-2^s^-1^; temperature (day/night) 25/20°C; RH 70 to 80%.

At the end of the growing period (27 days), Fe-deficient tomato plants clearly showed visible symptoms of Fe deficiency yellowing of the fully expanded apical leaves and proliferation of lateral roots and root hairs and increase in the diameter of the sub-apical root zone. 24 hours before harvesting, all nutrient solutions (both for Fe-deficient and Fe-sufficient plants) were renewed and the pH was adjusted to 7.5 with 10 mM Hepes-KOH. The pH of the growing medium was adjusted to this value to mimic as close as possible the conditions that are occurring in Fe-deficiency-inducing soil. However, in order to favour an equilibrate development of tomato plants growing in the Fe-free nutrient solution, the exposure to the pH of 7.5 was limited to the last two days.

Roots of Fe-deficient and Fe-sufficient tomato plants (27 d-old) were harvested five hours after the beginning of light phase. The collected roots were immediately frozen in liquid nitrogen and stored until further processing at -80°C. The collection was repeated in three independent cultivations and the roots from six plants were pooled for each treatment.

### Ferric-chelate reduction

To determine the root capacity to reduce Fe(III)-EDTA, accordingly to Pinton et al. [89] roots of a single intact (Fe-sufficient or Fe-deficient) tomato plants were incubated in the dark at 25°C for 60 min in 50 mL of an aerated solution containing CaSO_4 _0.5 mM, BPDS 0.5 mM, Hepes-KOH 10 mM (pH 5.5) and 0.25 mM of Fe(III)-EDTA. Thereafter, the absorbance of the solutions at 535 nm was measured at intervals of 15 min and the amount of Fe(III) reduced calculated by the concentration of the Fe(II)-BPDS_3 _complex formed, using an extinction coefficient of 22.1 mM^-1 ^cm^-1^.

### Microarray analysis

Transcriptional analysis was carried out using a Combimatrix [24], produced by the Plant Functional Genomics Center, University of Verona [90]. The chip (TomatoArray2.0) carries 25,789 nonredundant probes (23,282 unique probes and 2,507 probes with more than one target) randomly distributed in triplicate across the array, each comprising a 35-40-mer oligonucleotide designed using the program oligoarray 2.1 [91]. The source of sequence information included tentative consensus sequences (TCs) derived from the DFCI Tomato Gene Index [92] Release 12.0 and expressed sequence tags. Eight bacterial oligonucleotide sequences provided by CombiMatrix, 8 probes designed on 8 Ambion spikes and 40 probes based on *Bacillus anthracis, Haemophilus ducreyi *and *Alteromonas phage *sequences were used as negative controls. Complete description of chip is available at the Gene Expression Omnibus [93] under the series entry (GPL13934).

Total RNA was isolated using the Spectrum™ Plant Total RNA kit (Sigma-Aldrich) and quantified by spectrophotometry using NanoDrop™ 1000 Tem Scientific). RNA quality was evaluated using Agilent 2100 Bioanalyzer (Agilent). Total RNA (1 μg) was amplified and labelled using the RNA ampULSe kit (Kreatech). After checking the quantity and quality of aRNA by spectrophotometry using NanoDrop™ 1000 (Thermo Scientific) and the quality subsequent labelling, 4 μg of labelled aRNA was hybridized to the array according to the manufacturer's recommendations [24]. Pre-hybridization, hybridization, washing and imaging were performed according to the manufacture's protocols. The array was scanned with an Axon GenePix^® ^4400A scanner (MDS Analytical Technologies).

Analysis of raw data was performed using the open source software of the Bioconductor project [94,95] with the statistical R programming language [96,97]. Background adjustment, summarization and quantile normalization were performed using limma package [25]. Differentially expressed probes were identified by linear models analysis [25] using limma package and applying Bayesian correction, adjusted p-value of 0.05 and a |FC| ≥ 2. All microarray expression data are available at the Gene Expression Omnibus [93] under the series entry (GSE31112). Genes were grouped in main functional categories according to the "biological" terms of the Gene Ontology [27] assigned to each tomato TC or EST (Release 12.0) on the basis of the results of BlastP analysis [98] against the UniProt database [26] (Additional file 1). Genes without significant BlastP results were classified as "no hits found" (Evalue < 1e-8; identity > 40%).

### Real-time RT-PCR experiments

0.5 μg of total RNA (checked for quality and quantity using a spectrophotometer NanoDrop™ 1000 (Thermo Scientific), followed by a migration in an agarose gel) of each sample was retrotranscribed using 1 pmol of Oligo d(T)23VN (New England Biolabs, Beverly, USA) and 10 U M-MulV RNase H for 1 h at 42°C (Finnzymes, Helsinki, Finland) following the application protocol of the manufacturers. After RNA digestion with 1 U RNase A (USB, Cleveland, USA) for 1 h at 37°C, gene expression analyses were performed by adding 0.16 μl of the cDNA to the realtime PCR complete mix, FluoCycleTM sybr green (20 μl final volume; Euroclone, Pero, Italy), in a DNA Engine Opticon Real-Time PCR Detection (Biorad, Hercules, USA). Specific primers (Tm = 58°C) were designed to generate 80-140 bp PCR products (Additional file 2). Three genes were used as housekeeping to normalized the data: *elongation factor 1-alpha EF1a *(X14449; TC203463; forward: 5'- TGGATATGCTCCAGTGCTTG-3'; reverse: 5'-TTCCTTACCTGAACGCCTGT-3'), *histone H1 *(AJ224933; TC192148; forward: 5'- CAAAGGCCAAAACTGCTACC-3'; reverse: 5'-AGGCTTTACAGCTGCTTTCG-3') and *ubiquitin Ubi3 *(X58253; TC196208; forward: 5'-AGCCAAAGAAGATCAAGCACA-3'; reverse: 5'-GCCTCTGAACCTTTCCAGTG-3'). Each Real-Time RT-PCR was performed 4 times on 3 independent experiments; analyses of real-time result were performed using Opticon Monitor 2 software (Biorad, Hercules, USA) and R [93] with the qpcR package [99]. Efficiencies of amplification were calculated following the authors' indications [100]: PCR efficiencies were 99.15%, 89.16% and 87.25%, for *EF1a, H1 *and *Ubi3 *genes, respectively. The efficiencies for TC191581, TC192292, TC194139, TC216882, TC205660, TC208376 and NP796451 were respectively 94.75, 85.03, 98.92, 91.55, 92.70, 96.61 and 92.43%. The reported Real time data were normalized on the *EF1a *gene. Gene expression data were illustrated considering the differences in the amplification efficiency of PCR and using the gene expression levels in roots of Fe-sufficient plants as reference; applying the following formula:

$$gene\;\text{exp}\;ression\;\;\;x_{-Fe}=\frac{{\left(2\;\times E_{x}\right)}^{\left[c_{t}\left(x+Fe\right)-c_{t}\left(x-Fe\right)\right]}}{{\left(2\;\times E_{y}\right)}^{\left[c_{t}\left(y+Fe\right)-c_{t}\left(y-Fe\right)\right]}}$$

Where: E_x or y _is the percentage value of PCR efficiency for the amplification of the gene x or y, respectively; C_t_(x_+Fe_) Ct for the control treatment (+Fe) and the considered gene (x); *C_t_*(x_-Fe_); Ct for the treated roots (-Fe) and the considered gene (x); *C_t_*(y_+Fe_) Ct for the control treatment (+Fe) and the housekeeping gene (y); *C_t_*(y_-Fe_) Ct for the treated roots (-Fe) and the housekeeping gene (y).

## Competing interests

The authors declare that they have no competing interests.

## Authors' contributions

AZ and LZ made a substantial contribution to data collection and interpretation and manuscript drafting. NT participated in the project's design, data analysis and manuscript revision. MP critically revised the manuscript. RP contributed to data interpretation and critically revised the manuscript. ZV contributed to data interpretation and manuscript writing. SC participated in the project's design and coordination and critically revised the manuscript. All authors read and approved the final manuscript.

## Supplementary Material

Additional file 1 **Functional annotation of 97 differentially expressed transcripts**. ProbeID. Fold change. adjusted p-value. reference Tentative Consensus sequence in DFCI Tomato Gene Index (Release 12.0) http://compbio.dfci.harvard.edu/cgi-bin/tgi/gimain.pl?gudb=tomato. description. UniProtID http://www.uniprot.org/. Biological process GO term and E-value are reported for each probe. Click here for file

Additional file 2 **Primer sequences of Real-time RT-PCR experiment**. TC ID, description and sequences of forward and reverse primers are reported. Click here for file
